# Supplementary material for: Microplastic Distribution Characteristics and Sources on Beaches That Serve as the Largest Nesting Ground for Green Turtles in China
Source: Toxics. 2024 Jan 28;12(2):109. doi: 10.3390/toxics12020109 (PMC10891660; doi:10.3390/toxics12020109)
Supplement: Supplementary file 1 [file toxics-12-00109-s001.zip › toxics-2756909-supplementary.pdf]

Supplementary Information

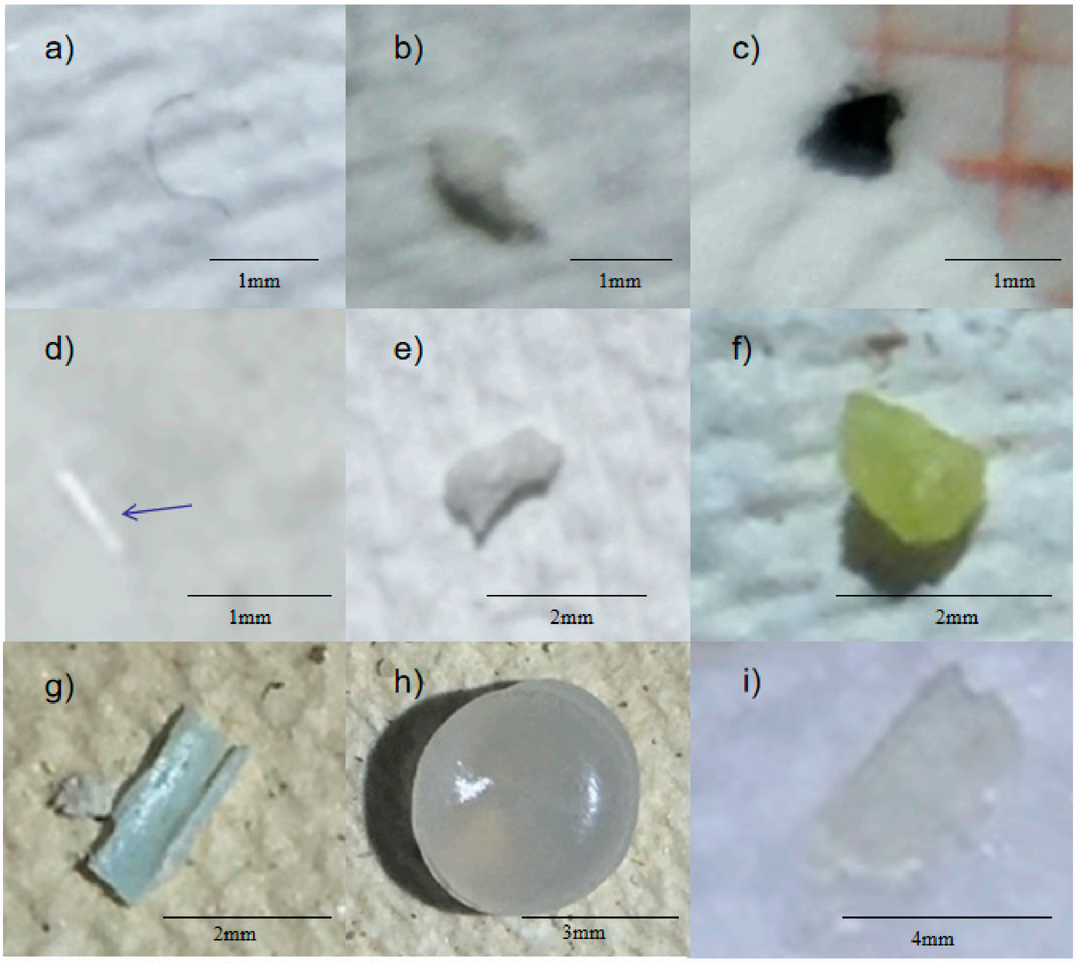

Figure S1. Examples of microplastics found in this study. a) Green fiber (PE); b) white foam (PS); c) black plastic block (PE); d) white film (PP); e) white foam (PS); f) yellow plastic block (PP); g) blue plastic block (PP); h) white microbeads (PE); i) white plastic block (PE).

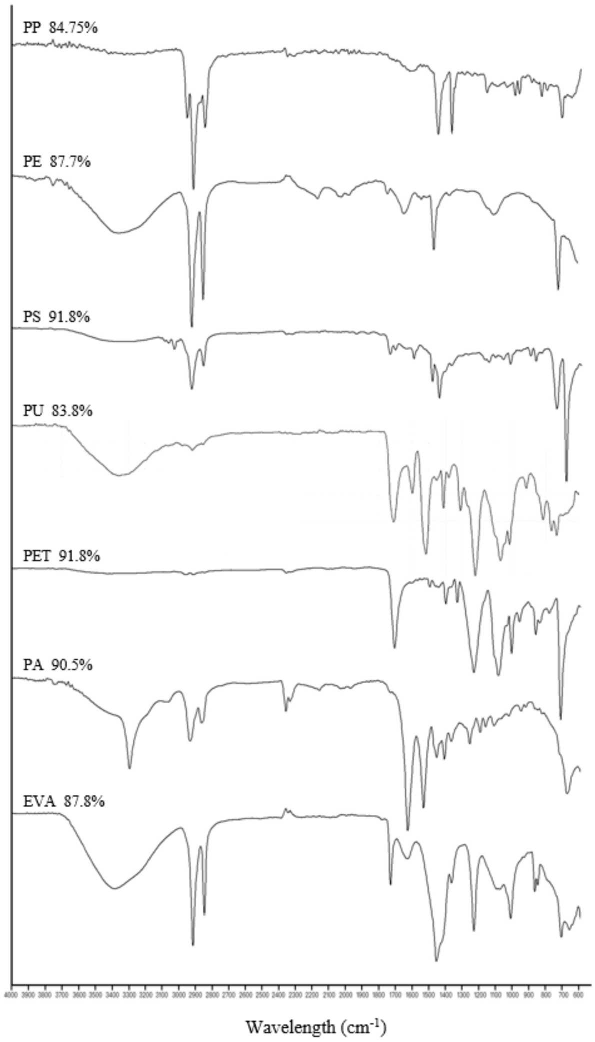

Figure S2. Typical spectrum of microplastic and microplastic components. Note：The percentage after the ingredient name indicates how similar the spectrum of the plastic ingredient is to the standard spectrum.

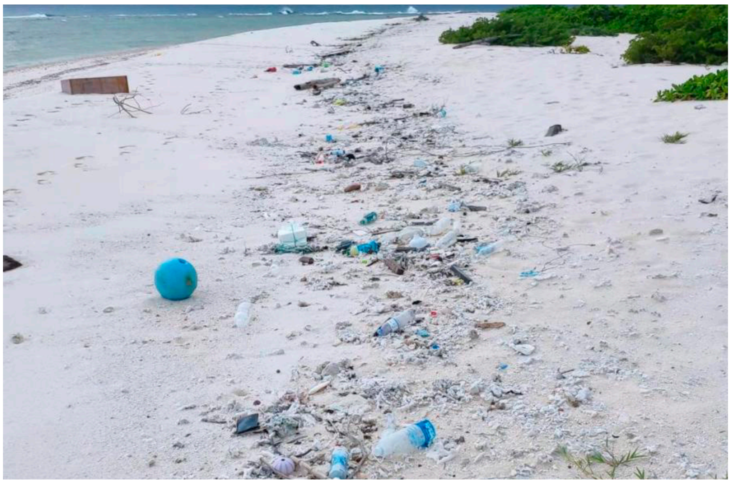

Figure S3. Plastic debris gathering in the supratidal zone on North Island, Qilianyu.

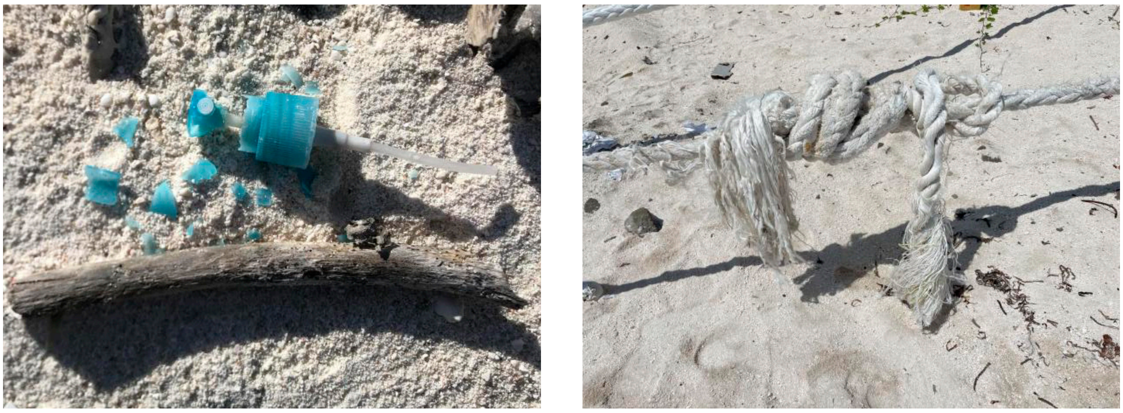

Figure S4. The phenomenon of microplastic shattering on the beach.
